# Healthcare professionals’ perspective on delivering personalised and holistic care: using the Theoretical Domains Framework

**DOI:** 10.1186/s12913-022-07630-1

**Published:** 2022-03-01

**Authors:** Eunice Wong, Felix Mavondo, Lidia Horvat, Louise McKinlay, Jane Fisher

**Affiliations:** 1 Monash Sustainable Development Institute, BehaviourWorks Australia, Monash University, Melbourne, Australia; 2 School of Public Health and Preventive Medicine, Monash University, Melbourne, Australia; 3 Department of Marketing, Monash University, Melbourne, Australia; 4 Safer Care Victoria, Department of Health Victoria, Melbourne, Australia

**Keywords:** Person-centred care, Theoretical Domains Framework, Behaviour change

## Abstract

**Background:**

Interventions to improve personalised and holistic care delivery by healthcare professionals are more likely to be effective if they target the factors influencing specific behaviours. This study reports on the development and testing of a questionnaire to identify perspectives of healthcare professionals’ personalised and holistic care behaviours based on the Theoretical Domains Framework.

**Methods:**

The study was conducted in public health services in Victoria, Australia. The questionnaire was developed and pilot-tested with behaviour change researchers and healthcare professionals. Doctors, nurses and midwives were recruited via notices and email invitations from Safer Care Victoria's website and mailing lists of healthcare professionals and invited to completed the questionnaire online (hosted on Qualtrics). Health services administrators and allied health professionals were excluded from the study. Confirmatory factor analysis was undertaken to generate the model of best fit and group differences were tested using univariate tests.

**Results:**

One hundred and four healthcare professionals from public health services in Victoria, Australia, completed the 39-item questionnaire focusing on specific personalised and holistic care behaviours. The final model consisted of 13 factors and 39 items, and CFA produced an acceptable fit, as well as adequate levels of discriminant validity and internal consistency (α = 0.60 to 0.84). Seven domains, “social influence”, “motivation & goals”, “environmental context and resources’, “skills”, ‘beliefs about consequences”, “behaviour regulation” and “nature of behaviour” were identified. Significant differences in the factors influencing these behaviours were found in groups with different years of experience and role seniority. These findings suggest that future interventions need to be targeted to specific groups.

**Conclusion:**

This study identified the specific behaviours and the factors associated with performance of personalised and holistic care among healthcare professionals. The findings suggest several interventions and policy functions may be taken to improve personalised and holistic care.

**Supplementary Information:**

The online version contains supplementary material available at 10.1186/s12913-022-07630-1.

## Background

Increasingly, policymakers, researchers and practitioners recognise the need to shift from the paternalistic and service-centric model of ‘physician knows best’ to a more collaborative and consultative approach with patients about their needs and experiences as users of health services. The focus is now on partnering with patients and their families in designing care, information and care pathways that fit the patient’s needs. This patient-centred care approach is associated with increased adherence and clinical benefits [1, 2] and reduced unnecessary health service visits [3, 4].

The Australian Commission on Safety and Quality in Health Care in Australia introduced the National Safety and Quality Health Service (NSQHS) standards (second edition) [5] in 2017 with a focus on partnering with patients. Following this, the state health authorities developed additional frameworks and guidelines to support health services to meet these standards. In the state of Victoria, Safer Care Victoria, in the Department of Health (DHS), developed a new framework ‘Partnering in Healthcare’, focusing on five key areas: ‘personalised and holistic care’, ‘effective communication’, ‘equity and inclusion’ ‘shared decision-making’ and ‘working together’ as a guide for health services in their work to improve patient experience and outcomes [6]. Safer Care Victoria worked with the public health services and ‘personalised and holistic care’ was one of the areas identified for implementation.

Despite all these initiatives, there is little evidence that patient experiences of care have improved significantly. A recent study in Victoria found that despite efforts by health services to improve the care delivered, patient experience scores have remained at 93%, below the target of 95%. It was found that more focus on dignity and respect; and emotional support could improve overall patient experience [7].

Interventions have typically been developed without a clear articulation of the theory of change [8, 9], often addressing the challenges with training or education, rather than using theory to understand the barriers and levers for behaviour change [10, 11].

To identify healthcare professionals’ behaviours associated with personalised and holistic care. The ‘Partnering in Healthcare’ framework [12] first defines it as understanding the whole person (or family); physical, cultural, social context, and differences in person’s health, wellbeing and safety. The elements include putting people and families at the centre of care, providing emotional support and empathy, involving family and friends and showing compassion and respect. A recent qualitative study identified specific, measurable actions associated with these concepts, particularly behaviours that i) build relationships such as taking time with patients, active listening, expresses caring and empathy; ii) personalised care practices such as the inclusion of family, knowing the patient, eliciting and respecting patients’ values [13]. The second step is understanding the factors that influence the performance of behaviours associated with personalised and holistic care.

Theoretical models and frameworks for behaviour change of healthcare professionals have been proposed for clinical practices for the management of different conditions [10, 14–16]. Many of which identify individual factors (knowledge, skills, self-efficacy); social factors (social support, group norms); and environmental factors (resources, organisational climate). The Theoretical Domains Framework (TDF) [17, 18] proposes an integrated theoretical framework where 128 commonly related constructs are grouped into 12 domains to aid in identifying influences on the behaviour. Subsequently, the TDF authors further expanded on their work with the behaviour change wheel (BCW) [19] to provide a guide to identifying interventions to target the factors influencing the behaviours.

Research using TDF to understand and change healthcare professionals’ behaviours has mostly been qualitative [14, 17, 20]. This has provided a rich detailed understanding of the factors for change, but these studies cannot be generalised. There are a small number of studies where TDF has been used to develop questionnaires with selected domains and constructs that were assessed as relevant to the respective contexts examining healthcare professionals’ behaviours towards clinical practice guidelines [21], patient safety [22] and smoking [23]. From the above mentioned TDF questionnaire studies, it is observed that not all domains and constructs are comprehensively covered, prioritisation was conducted based on the context of the target behaviour, admittedly respondent fatigue may be a key consideration. To the best of our knowledge, the application of TDF to the development of a questionnaire to understand healthcare professionals’ personalised and holistic care behaviours does not exist in the literature. The use of a questionnaire based on the TDF can identify key factors for behaviour change that can be applied to all health services. This allows reaching out to a larger sample and providing robust findings for policy interventions.

The aim was to develop and test a questionnaire using the TDF to understand healthcare professionals’ perspectives on performing personalised and holistic care behaviours.

## Methods

### Measure

#### Development of the views and experiences of personalised and holistic care questionnaire

##### Consultation with Safer Care Victoria to identify target behaviours

Consultation sessions with Safer Care Victoria’s Partnering in Healthcare framework team were conducted to identify target behaviours. As personalised and holistic care is multifaceted, Safer Care Victoria’s team highlighted that in practice, identifying and prioritising a single behaviour was inadequate and not representative. Other questionnaire studies guided by TDF addressed this challenge in the following ways i) use of broad non-behavioural statements such as ‘follow/use current care guidelines’ in studies [24–26], ii) restrict and prioritised only one behaviour as in the case of a study exploring clinicians performing patient safety behaviour [22] and iii) identify a set of behaviours described in respective care guidelines to be randomised for each item in studies [23, 27]. Based on the feedback on the context and inputs from the analysed qualitative data from Safer Care Victoria’ framework development team six specific behaviours; 1) speak with patients about their anxieties/fears with their health condition, 2) ask patients about the expectations of their care, 3) demonstrate respect and courtesy, 4) discuss patients’ care goals with them, 5) review patients’ medical information before meeting them and 6) introduce yourself to the patient and family, were identified to be randomised for item development to provide a more comprehensive representation of personalised and holistic care behaviours.

##### Items development

The questionnaire items were developed based on the original 12 domain version of the TDF assessing the domains of behaviour change [26]. The findings from the regular update and adaptation of the framework to specific behaviours studied [24–26, 28], suggests that the original 12-domain version of the TDF instead of the updated 14-domain version might be more applicable for questionnaire design and only the domains relevant to the behaviours studied should be used.

In the development of the items, the authors reviewed and considered the relevance and application of each of the 12 domains of the TDF in clinical settings. Finally, two domains “Emotion” and “Memory, attention and decision processes” were excluded, based on the consideration that these domains may be more applicable to studying patient self-management behaviours but not as relevant to healthcare professionals’ performance of personalised and holistic care behaviours. In addition, consideration was given to the length of the questionnaire with a view to minimise respondent fatigue. The authors debated and prioritised each construct through a process of elimination, including only keeping key constructs that were most relevant and observable and would not suffer from potential social desirability. This prioritisation was necessary as it was not feasible to ask questions for all theoretical constructs grouped under the ten domains.

The final questionnaire included 39 items assessing ten domains and their related key constructs that were relevant to healthcare professionals’ personalised and holistic care behaviours (See Table 1). To assure the reliability of measures, a minimum of three items for each construct was developed. As this study is guided by the TDF, each item was developed to measure the corresponding theoretical construct, the authors with their extensive experience in clinical psychology, public health and questionnaire development conducted rounds of revisions to carefully construct the items to align to the theoretical definitions of the constructs.

**Table 1:** TDF Domains and Description of Constructs

**TDF (10 domains)**	**Constructs (13)**	**Brief description of constructs**
Knowledge	1.Knowledge	Know how to speak and interact with patients on their care
Skills (Cognitive and Interpersonal)	2.Skills	Interpersonal skills and consideration from patients’ point of view
Behavioural regulation	3.Self-monitoring	Skills needed to monitor the behaviours
	4.Action planning	Skills needed to plan the behaviours
Nature of behaviour	5.Automaticity	Performing the behaviours without thought routines, habits
Environmental context and resources	6.Resources/materials	The extent availability, physical or resource factors affect the delivery of patient-centred care
Social influences	7.Social support	Healthcare professionals (HCP) can count on their colleagues when there are problems
	8.Subjective norm	HCP think that colleagues who matter to them approve of their behaviours
Professional/social role and identity	9.Professional role	HCPs view it as their professional role to perform these behaviours
Beliefs about capabilities	10.Self- efficacy	HCPs’ self-belief in their ability to perform those behaviours
Motivation and goals	11.Priority	HCPs’ viewed it as important to perform these behaviours in comparison with other tasks or behaviours
Beliefs about consequences	12.Reinforcement	HCPs’ are recognised or not when they performed these behaviours
	13.Outcome expectations	HCP think that there is a worthwhile outcome from their performance of these behaviours

Further considerations in the development of the items were to word them to include targets, actions, contexts and time, following Fisbein and Ajzen’s approach [29] while retaining the theoretical content in each item. To reduce and address the social desirability bias in self-report questionnaires [30], indirect questions rather than direct questions were included as much as possible [31]. The items were also adapted from i) previous studies [25, 26, 32] that used the TDF in their questionnaires to understand healthcare professionals’ behaviours or change behaviours in healthcare settings, ii) Safer Care Victoria’s prioritised personalised and holistic care behaviours obtained from the development of the ‘Partnering in Healthcare’ framework [12]. This questionnaire was developed in collaboration with Safer Care Victoria and tested as part of their implementation work.

The demographic questions included in the questionnaire were age, gender, professional role, health service type (metropolitan, regional or rural), years of working experience and work status (full-time, part-time or casual).

##### Piloting of questionnaire

Piloting was undertaken in two rounds. The first round was conducted with four behaviour change researchers with postgraduate training in behavioural science to determine whether items were worded clearly, had face validity for the constructs being measured and included target, action, context and time. They were also asked to pilot the functionality of the online survey platform (Qualtrics) hosting the questionnaire. Based on their feedback, amendments were made to the wordings of the items before further piloting.

The final round of piloting was undertaken with a varied group of seven healthcare professionals, nurses, midwives, and doctors with clinical and public health experience on the comprehension, face validity and the context of the items. Amendments to the final wordings of the items were made based on the feedback.

An online version of the questionnaire was used to measure the healthcare professionals’ self-reported performance of personalised and holistic care behaviours. Each item was assessed on a 7-point Likert type scale ranging from 1 = *Never* or *Strongly Disagree* to 7 = *Always* or *Strongly Agree.* (See Additional file1 for questionnaire items).

### Participants

#### Inclusion and exclusion criteria

Only participants who identified as doctors, nurses and midwives aged at least 18 years and working in public health services in the Australian state of Victoria were eligible to participate.

#### Data collection procedure

The online questionnaire was undertaken between 3 Aug and 8 Oct 2020. Safer Care Victoria disseminated the information of the study and hyperlink to the questionnaire (hosted on Qualtrics platform) on their website, Linkedin and Twitter platforms. Further dissemination was conducted by Safer Care Victoria via targeted emails to subscribers to their clinical networks e-newsletter and mailing lists of patient experience and partnering in healthcare coordinators in Victoria’s public health services. The emails and posts invited interested participants to access the hyperlink to provide consent and participate in the questionnaire. No incentive was offered for completion, and participation was anonymous and entirely voluntary.

### Data analysis

#### Discriminant validity and internal consistency reliability

The questionnaire was developed with a focus on ten domains with 13 specific constructs based on the Theoretical Domains Framework (TDF). Confirmatory factor analysis was used to identify the number of constructs in the questionnaire responses. Cronbach alpha was calculated to assess the internal consistency/reliability of the constructs. Convergent and discriminant validity were assessed through the calculation of the average variance extracted (AVE) and correlating the latent constructs.

#### Group differences

One-way ANOVA and t-tests were used to examine the perspectives of the professional group, role seniority, years of work experience, gender and type of health service category. The data were compiled and analysed using IBM SPSS V.26 and AMOS V.26.

#### Ethics approval

Ethics approval for this study was provided by the Monash University Human Research Ethics Committee (Project ID 2020–23,630-47,331).

## Results

In total, 104 healthcare professionals contributed data. The sample included 48 (46%) nurses, 33 (32%) doctors, 19 (18%) midwives and four (4%) who did not provide details regarding their profession. The average age of the participants was 45.62 years (SD = 11.10), and there were 86 females (82.7%) and 18 males (17.3%). Most respondents worked in metropolitan health services (67.5%), with 20% working in regional health services and 12.5% in rural health services. On average, the participants had worked in their profession for 18.25 years (SD = 11.90).

### Analysis

#### Confirmatory factor analysis

A 13-factor model was specified and evaluated with the sample using confirmatory factor analysis, employing maximum likelihood estimation, in IBM AMOS 26. The model fit was evaluated using a range of goodness of fit indices. The data did not fit the 13-factor model well; upon inspections of the modification indices (M.I.s) and item content, the factors knowledge and skills did not have discriminant validity. Nevertheless, as the factors knowledge and skills are separate domains in the TDF, a final 13 factor model was retained with the rest of the factors showing adequate measures of fit (see Table 2).

**Table 2:** Confirmatory factor analysis model fit indices

TDF Domains	Constructs	${x}^{2}$	*df*	CMIN/*df*	GFI	NFI	CFI	RMSEA
Knowledge	Knowledge	20.97	7	2.99	.943	.936	.955	.100
Skills	Skills							
Behavioural regulation & Nature of behaviour	Self-Monitoring	9.89	11	0.90	.975	.969	1.00	.001
	Action Planning							
	Automaticity							
Environmental context and resources & Social influences	Resources	26.9	15	1.79	.946	.922	.962	.080
	Social Support							
	Subjective Norm							
Professional role & Self-efficacy	Professional Role	7.18	4	1.79	.971	.949	.976	.080
	Self-efficacy							
Motivation & Goals	Action Planning	9.34	8	1.17	.972	.944	.991	.040
	Priority							
Belief about consequences	Outcome Expectation	5.13	4	1.28	.981	.959	.990	.052
	Reinforcement							

#### Discriminant validity and internal consistency reliability

The constructs of ‘subjective norm’, ‘self-efficacy’, ‘automaticity’ and ‘action planning’ were not found to show adequate discriminant validity, as their square root of average variance extracted (AVE) were higher than the correlation between the factors [33]. Chi-square difference test was used to assess discriminant validity where the correlation between latent factors was larger than the square root of AVE. In all cases, discriminant validity was supported (see Table 3). The internal consistency reliabilities were calculated using Cronbach’s alpha and values ranged from 0.60 to 0.76.

**Table 3:** Correlation of factors influencing personalised and holistic care behaviours

Correlation matrix of personalised care behaviours													
Constructs	1	2	3	4	5	6	7	8	9	10	11	12	13
1. Social Support	**0.65**												
2. Reinforce	.40^**^	**0.66**											
3. Priority	.10	.08	**0.68**										
4. Subjective Norm	.66^**^	.31^**^	.09	**0.74**									
5. Self Monitoring	.40^**^	.28^**^	.42^**^	.51^**^	**0.73**								
6. Resources	.63^**^	.38^**^	.17	.69^**^	.54^**^	**0.57**							
7. Knowledge	.36^**^	.43^**^	.31^**^	.54^**^	.68^**^	.59^**^	**0.56**						
8. Skills	.45^**^	.45^**^	.34^**^	.56^**^	.62^**^	.55^**^	.87^**^	**0.44**					
9. Self-Efficacy	.54^**^	.55^**^	.29^**^	.48^**^	.70^**^	.58^**^	.76^**^	.77^**^	**0.66**				
10 Outcome Expectation	.38^**^	.39^**^	.30^**^	.43^**^	.41^**^	.37^**^	.64^**^	.65^**^	.59^**^	**0.78**			
11. Professional Role	.45^**^	.27^**^	.26^**^	.47^**^	.52^**^	.38^**^	.59^**^	.57^**^	.62^**^	.51^**^	**0.69**		
12. Automaticity	.54^**^	.50^**^	.36^**^	.54^**^	.61^**^	.60^**^	.81^**^	.77^**^	.82^**^	.73^**^	.64^**^	**0.72**	
13. Action Planning	.65^**^	.40^**^	.40^**^	.51^**^	.67^**^	.63^**^	.64^**^	.69^**^	.71^**^	.58^**^	.50^**^	.76^**^	**0.72**
Cronbach Alpha	0.67	0.68	0.71	0.69	0.62	0.60	0.76	0.68	0.68	0.73	0.65	0.69	0.76
Composite Reliability	0.67	0.69	0.71	0.70	0.69	0.59	0.79	0.70	0.70	0.76	0.65	0.69	0.76
Skewness	-0.19	-0.07	0.34	-0.65	-0.71	-0.65	-0.71	-0.54	-0.35	-0.93	-1.24	-0.72	-0.44
Kurtosis	-0.61	-0.97	-0.43	-0.44	-0.12	-0.03	0.06	-0.22	-0.74	0.44	1.43	-0.08	-0.54

Legend: * = *p* < .05, ** = *p* < .01 Figures in bold on the diagonal are square root of AVE, figures below the diagonal are Pearson’s correlations

#### Personalised and holistic behaviours

The mean scores for each of the constructs are presented in Table 4. The values for all the constructs indicated relatively high mean scores (above 5 on a 7-point scale) with the exception of the ‘priority’ (*x* = 3.84) construct (for description, see Table 1). In this study, the cut-off means scores for each of the constructs indicating 95% (*x* ≥ 6.65), 85% (*x* ≥ 5.95) and 75% (*x* ≥ 5.25) agreement were used. This was determined to align with the performance standards of quality care for public health services set by the Department of Health Services (DHS) in Victoria [34]. For example, public health services are expected to attain 100% on the timely access to care standard, 95% on overall patient experience of care and 75% on the coordination of care (see Table 4). The mean scores of all constructs fall below the 95% DHS standard level. The constructs falling below 85% are' action planning', 'automaticity', 'reinforcement', 'social support', 'resources', 'subjective norm' and ' Skills'. Only the behaviour ‘priority’ was below 75%.

**Table 4:** Mean Score of Constructs, TDF domains and Levels of Self-reported Attainment

**TDF Domains**	**Constructs of Personalised and Holistic care extracted**	**(*n* = 104)**			**Healthcare professionals self-reported attainment levels**		
		**Mean**		**(S.E.)**			
					**75%**	**85%**	**95%**
Social influences	1. Social Support for staff	5.67	(.09)			++	+
	2. Subjective Norm	5.77	(.11)			++	+
Motivation & goals	3. Priority	3.84	(.12)		+++		+
Behavioural regulation	4. Self-Monitoring	6.24	(.07)				+
	5. Action Planning	5.43	(.10)			++	+
Environmental context & resource	6. Resources	5.67	(.09)			++	+
Knowledge	7. Knowledge	6.00	(.08)				+
Skills	8. Skills	5.92	(.08)			++	+
Beliefs about capabilities	9. Self-efficacy	6.04	(.07)				+
Beliefs about consequences	10. Outcome Expectation	5.98	(.09)				+
	11. Reinforcement	5.61	(.09)			++	+
Professional role	12. Professional Role	6.04	(.10)				+
Nature of behaviour	13. Automaticity	5.56	(.11)			++	+

Legend: + = below 95%; ++ = below 85%; +++ = below 75%

Further subgroup analyses by the professional group, role seniority, years of experience, gender and health service category variables were undertaken to examine any differences in the mean scores for each construct. No differences by gender and health service category were identified. However, significant differences were found across the professional groups (p < 0.01), as shown in Table 5. There was no difference found in the ‘social support’, ‘reinforcement’, ‘subjective norm’, ‘resources’ and ‘knowledge’ behaviours. Respondents who identified as midwives reported higher mean scores in the ‘self-efficacy’, ‘professional role’, ‘automaticity’ and ‘action planning’ behaviours compared to those identified as nurses or doctors. Respondents identified as nurses reported the lowest mean scores in the ‘priority’ behaviours compared to the other groups.

**Table 5:** One-way analyses of variance for the effects of professional group on personalised and holistic care constructs

One-Way ANOVA	Professional Group								
TDF constructs	(A)			(B)		(C)		F- ratio	Differences
	Nurse (*n* = 48)			Doctor (*n* = 33)		Midwife (*n* = 19)			
	M	(S.E.)		M	(S.E.)	M	(S.E.)		
Social Support	5.71		(.13)	5.45	(.15)	6.02	(.15)	2.62	
Reinforcement	5.51		(.12)	5.48	(.18)	5.98	(.21)	2.07	
**Priority**	**3.39**		**(.14)**	**4.07**	**(.21)**	**4.51**	**(.30)**	7.95**	**A < B, C**
Subjective Norm	5.61		(.15)	5.95	(.17)	5.87	(.29)	1.10	
Self-Monitoring	6.10		(.12)	6.15	(.12)	6.61	(.11)	3.54*	C > A, B
Resources	5.60		(.15)	5.54	(.13)	5.98	(.17)	1.66	
Knowledge	5.85		(.12)	6.01	(.13)	6.25	(.14)	1.87	
Skills	5.69		(.12)	5.95	(.13)	6.28	(.13)	4.29*	C > A
Self-efficacy	5.99		(.11)	5.80	(.12)	6.49	(.08)	6.24**	C > A, B
Outcome Expectation	5.76		(.14)	5.98	(.14)	6.39	(.13)	3.72*	C > A
Professional Role	5.75		(.17)	6.15	(.17)	6.45	(.13)	3.67**	C > A
Automaticity	5.33		(.18)	5.42	(.17)	6.29	(.13)	5.89**	C > A, B
**Action Planning**	**5.24**		**(.16)**	**5.29**	**(.14)**	**6.14**	**(.10)**	7.18**	**C > A, B**

Legend: * = *p* ≤ .05, ** = *p* ≤ .01 Figures in bold are those constructs that are below 85% with group differences

The association between role seniority and the mean scores of personalised and holistic care constructs is shown in Table 6. Role seniority was categorised based on the job titles reported by respondents; i) Junior nurse (registered nurse, enrolled nurse), ii) Senior nurse (clinical nurse specialist, nurse unit manager, clinical educators, nurse practitioner), iii) Midwife, iv) Junior doctor (resident/intern doctor, registrar) and v) Senior doctor (consultant, senior consultant). There were insufficient data to compare group differences for junior doctors. Besides the effect of the midwife group, significant differences (p < 0.01) was found where senior nurses reported higher score in ‘self-monitoring’ than junior nurses and senior doctors. Another difference is that the junior nurse group reported lower scores in ‘priority’ than senior doctor and midwife groups.

**Table 6:** One-way Analyses of Variance for the Effects of Role Seniority on Personalised and Holistic Care Constructs

One-Way ANOVA	Role Seniority									
TDF constructs	(D)		(E)		(F)		(G)		F- ratio	Differences
	Junior Nurse		Senior Nurse		Senior Doctor		Midwife			
	(*n* = 33)		(*n = *15)		(*n* = 31)		(*n* = 19)			
	M	(S.E.)	M	(S.E.)	M	(S.E.)	M	(S.E.)		
Social Support	5.60	(.18)	5.96	(.17)	5.44	(.16)	6.02	(.15)	2.37	
Reinforcement	5.41	(.15)	5.73	(.24)	5.48	(.19)	5.98	(.21)	1.75	
Priority	3.16	(.16)	3.89	(.27)	4.05	(.21)	4.51	(.30)	6.86**	D < F, G
Subjective Norm	5.41	(.19)	6.07	(.15)	5.90	(.18)	5.87	(.29)	1.94	
Self-Monitoring	5.92	(.14)	6.50	(.18)	6.13	(.12)	6.61	(.11)	4.87**	E > D, F
Resources	5.40	(.20)	6.04	(.14)	5.52	(.14)	5.98	(.17)	3.04	
Knowledge	5.71	(.15)	6.16	(.19)	5.98	(.14)	6.25	(.14)	2.42	
Skills	5.62	(.14)	5.84	(.23)	5.91	(.14)	6.28	(.13)	3.07*	G > D
Self-efficacy	5.95	(.14)	6.09	(.18)	5.81	(.13)	6.49	(.08)	4.03**	G > D, F
Outcome Expectation	5.71	(.17)	5.87	(.25)	5.94	(.14)	6.39	(.13)	2.55	
Professional Role	5.52	(.21)	6.27	(.23)	6.15	(.18)	6.45	(.13)	4.44**	G > D
Automaticity	5.17	(.23)	5.70	(.27)	5.40	(.18)	6.29	(.13)	4.84**	G > D, F
**Action Planning**	**5.08**	**(.21)**	**5.58**	**(.23)**	**5.27**	**(.14)**	**6.14**	**(.10)**	5.92**	**G > D, F**

Legend: * = *p* ≤ .05, ** = *p* ≤ .01 Figures in bold are those constructs that are below 85% with group differences

The effect of years of experience, where respondents with less than ten years of experience reported lower scores in ‘subjective norm’ and ‘resources’ constructs than those with more years of experience is shown in Table 7.

**Table 7:** One-way analyses of variance for the effects of years of experience on personalised and holistic care constructs

One-Way ANOVA	Years of Experience							
TDF constructs	(H)		(I)		(J)		F- ratio	Differences
	≤ 10 years		11–25 years		> 25 years			
	(*n* = 37)		(*n* = 35)		(*n* = 32)			
	M	(SE)	M	(SE)	M	(SE)		
**Social Support**	**5.39**	**(.16)**	**5.93**	**(.13)**	**5.72**	**(.14)**	3.65*	**H < I**
Reinforcement	5.77	(.16)	5.50	(.15)	5.54	(.16)	0.85	
Priority	3.82	(.23)	3.96	(.22)	3.72	(.17)	0.34	
**Subjective Norm**	**5.30**	**(.20)**	**5.96**	**(.18)**	**6.11**	**(.12)**	6.23**	**H < I, J**
Self-Monitoring	6.04	(.14)	6.30	(.11)	6.41	(.12)	2.39	
**Resources**	**5.20**	**(.17)**	**5.89**	**(.11)**	**5.97**	**(.13)**	9.24**	**H < I, J**
Knowledge	5.86	(.14)	5.96	(.13)	6.22	(.13)	1.96	
Skills	5.88	(.13)	5.81	(.14)	6.07	(.13)	0.98	
Self-efficacy	5.99	(.12)	5.99	(.13)	6.16	(.12)	0.58	
Outcome Expectation	6.08	(.13)	5.86	(.17)	5.98	(.14)	0.58	
Professional Role	5.80	(.20)	6.24	(.17)	6.09	(.14)	1.77	
Automaticity	5.45	(.22)	5.54	(.17)	5.72	(.16)	0.53	
Action Planning	5.34	(.18)	5.40	(.16)	5.57	(.14)	0.50	

Legend: * = *p* ≤ .05, ** = *p* ≤ .01 Figures in bold are those constructs that are below 85% with group differences

## Discussion

Overall, there was a high level of self-reported attainment and regard for personalised and holistic care behaviours among the doctors, nurses and midwives in Victoria, Australia. The results support those observed in earlier studies [35, 36] of healthcare professionals’ acceptance of and efforts to change patient-centred practices. This study identified seven behavioural domains ‘environmental context and resources’, ‘skills’, ‘social influence’, ‘beliefs about consequences’, ‘behavioural regulation’, ‘nature of behaviour’ and ‘motivation and goals’ that are central to increased uptake of personalised and holistic care behaviours.

As found by others [37, 38], ‘skills’, ‘environmental’ (resources construct) and ‘motivation and goals’ (priority construct) domains are common barriers to changing healthcare professionals’ behaviours in the uptake of guidelines. The findings of this study lend further support to these previous findings. The ‘motivation and goals’ (priority construct), where participants rate the importance of performing personalised and holistic care behaviours compared to other tasks, had the lowest score suggesting a more substantial challenge to overcome than the rest. However, it could be the result of increased workload in the public health services. This is more so given that this study was conducted during the period of strict lockdown in Victoria to combat high rates of COVID-19 infections. Beyond the conventional limitations of capability, time and resources, earlier studies [10, 39] on healthcare professionals’ behaviour change had begun investigating other predictors of behaviour change and drawing explanation from behavioural theories.

The study’s findings of ‘social influence’, ‘beliefs about consequences’, ‘behavioural regulation’ as factors that influence healthcare professionals’ behaviours are significant in at least two major respects. First, to improve personalised and holistic care, there is a need to look beyond conventional challenges of capability, time and resources, as shown in earlier studies[10, 39, 40]. Second, there is potential for more improvement through promoting peer group norms and expectations, and reminders to adopt the desired behaviours within the context. Earlier studies found that when these factors were addressed and the behaviours became habitual, healthcare professional behaviours were changed [10, 41].

The group differences found in years of experience and role seniority indicate that when designing interventions to target the behavioural domains, there is a need to customise for those with ten or less years of experience, senior doctors and junior nurses. The reason for the differences is not clear or explored in this study, but it may be due to the level of autonomy and confidence in their professional practices.

### Strengths and limitations

To date, this is, to our knowledge, one of the first studies to use the TDF to quantify the behavioural domains in implementing personalised and holistic care by doctors, nurses and midwives. The identified domains from the TDF can be targeted in the design of interventions to improve personalised and holistic care. This study also analysed the mean scores for the constructs by benchmarking the performance standard (95%) required of health services instead of the usual criterion of five out of seven on a Likert scale. This resulted in the identification and prioritisation of TDF domains to focus on despite high scores overall. Personalised and holistic care is one aspect of quality of care, just like patient safety, where performance under 95% would be unacceptable and would call for urgent improvement.

Limitations of this study include the small sample size for the questionnaire and the model fit for the ‘knowledge’ and ‘skills’ constructs. These could be addressed in future studies with a larger sample size and revision of the questionnaire items in these constructs. While questionnaire design met the aims of this study, it has limited the scope of the TDF, as not all domains and theoretical constructs were comprehensively covered and tested. The findings of this study are limited to the domains and related constructs tested in the questionnaire and it is unknown if the untested domains are also relevant and to what extent healthcare professionals perform these behaviours.

### Implications for practice

For health service practitioners and policymakers, this study may be used to identify the influencing factors for personalised and holistic care that are not meeting the targeted standards. This allows for prioritisation of interventions. Next, there is a need to design and trial interventions tailored to the different healthcare professional groups in their context. This could be guided by theory-informed intervention functions and policy categories [17, 42–44].

### Implications for research and suggestions for future research

To add to the design of theory-informed interventions for practice, a summary table (see Table 8) below is adapted from the authors of behaviour change wheel [45] as a start to identify intervention functions and policy categories. Future research efforts could be focused on detailed mapping and evaluation of the intervention functions and policy categories for personalised and holistic care using this framework and the subsequent design of intervention strategies [46–48].

**Table 8:** Mapping of personalised and holistic care to intervention functions and policy categories using TDF and behaviour change wheel

COM-B	TDF domains	Brief description of domains	Intervention functions	Policy categories
Capability (Psychological)	Knowledge Skills	Know how to speak and interact with patients on their care Interpersonal skills and consideration from patients’ point of view	Education Training	Communication and marketing, guidelines
	Behavioural regulation	Skills needed to monitor and plan behaviours	Education, training, modelling, and enablement	Environment/social planning and service provision (introduction of new service or function)
	Nature of behaviour	Performing the behaviours without thought routines, habits	Training, environmental restructuring and enablement	Guidelines, regulation, environment/social planning, and service provision
Physical opportunity	Environmental context and resources	The extent availability, physical or resource factors affect the delivery of personalised and holistic care	Training, environmental restructuring and enablement	
Social opportunity	Social influences	Healthcare professionals (HCP) can count on their colleagues when there are problems	Restriction, environmental restructuring, modelling and enablement	Guidelines and environment/social planning
		HCP think that colleagues who matter to them approve of their behaviours		
Reflective motivation	Motivation and goals	HCP viewed it as important to perform these behaviours in comparison with other tasks or behaviours	Education, persuasion, incentivisation, modelling and enablement	Communication/marketing, guidelines, environment/social planning, service provision
Automatic motivation	Beliefs about consequences	HCPs’ are recognised or not when they performed these behaviours. HCP think that there is a worthwhile outcome from their performance of these behaviours	Training, incentivisation, coercion and environmental restructuring	

*Adapted from* Michie S, Atkins L, West R. (2014) The Behaviour Change Wheel: A Guide to Designing Interventions. London: Silverback Publishing

## Conclusion

This study contributes to understanding factors associated with personalised and holistic care among healthcare professionals in Victoria, Australia. The self-reported performance of personalised and holistic care behaviours was relatively high but still falls short of the 95% standard for overall patient experience set for health services. Although encouraging, further improvement is needed before personalised and holistic care is accorded the same consideration as patient safety in measuring care quality. As the way forward, future implementation and interventions can start by addressing the identified factors falling too far below the desired standards.

## Supplementary Information

**Additional file 1.**

## Data Availability

The datasets used and/or analysed during the current study are available from the corresponding author on reasonable request.

## Abbreviations

TDF: Theoretical Domains Framework
BCW: Behaviour Change Wheel
NSQHS: National Safety and Quality Health Service
CFA: Confirmatory factor analysis
AVE: Average variance extracted
DHS: Department of Health Services
