# Supplementary material for: Healthcare professionals’ perspective on delivering personalised and holistic care: using the Theoretical Domains Framework
Source: BMC Health Serv Res. 2022 Mar 1;22:281. doi: 10.1186/s12913-022-07630-1 (PMC8887936; doi:10.1186/s12913-022-07630-1)
Supplement: Supplementary file 1 — Additional file 1. [file 12913_2022_7630_MOESM1_ESM.docx]

Additional file 1

39-items Personalised and Holistic Care Survey mapped to TDF domains and related constructs

| **TDF V1 (12 domains)** | **Constructs** | **Personalised and Holistic Care** |
| --- | --- | --- |
|  |  | 39 items |
| D1 Knowledge | Procedural knowledge | 1. I am familiar with how to speak with patients about their anxieties/fears with their health condition 2. I ask patients about what they know about their health condition to reach a common understanding 3. I demonstrate respect for the patients and their families by introducing myself and my team. |
| D2 Skills (Cognitive and Interpersonal) | Skills | 1. I have been trained in discussing patients’ care goals with them to enable me to plan their discharge 2. I am able to acknowledge patients’ anxieties/fears about their health condition. 3. I am prepared for my discussions with patients and their families/carers by reviewing their medical information beforehand |
| D11 Behavioural regulation | Self-monitoring | 1. I consciously seek to demonstrate courtesy and respect to patients. 2. I check regularly with patients whether I have understood their anxieties/fears about their health condition. 3. I am aware of the need to introduce myself and my team to the patient and family at every new interaction. |
| D12 Nature of behaviour | Automaticity | 1. I routinely discuss with patients, their anxieties/fears about managing their care post-discharge. 2. I introduce myself to patients and their families is something I do without thinking. 3. Reviewing patients’ medical information before speaking with them is second nature to me |
| D8 Environmental context and resources | Resources/materials | 1. In the hospital I work in, it is standard practice for clinicians to routinely introduce themselves to the patients and their families/carers. 2. Despite time constraints on my work at the hospital, I am still able to speak to patients about their anxieties/fears about their health condition. 3. There are policies in the hospital I work in that encourage clinicians to discuss with patients on their expectations of care. |
| D9 Social influences | Social support | 1. My colleagues are supportive of my efforts to speak with patients about their anxieties and fears as part of providing personalised care 2. My colleagues are willing to listen to my concerns when I am unsure on the patients’ expectations of their care. 3. The team I work with is helpful in getting the patients’ medical information updated for review before I speak with patients. |
|  | Subjective norm | 1. My colleagues consider it very important that I speak with the family/carers of a patient as part of discharge planning 2. Most colleagues who opinions I value would approve of me speaking with patients about their expectations for care. 3. Colleagues whom I look up to, think that every patient should be treated with courtesy and respect. |
| D3 Professional/social role and identity | Professional role | 1. I consider it my professional responsibility to find out what patients expect from their post-discharge care so we can jointly work towards it. 2. It is part of my professional role, to be prepared before speaking with patients about their discharge plan by reviewing their medical information beforehand. 3. Introducing myself and my team to patients and their families/carers is what every health care professional should do. |
| D4 Beliefs about capabilities | Self- efficacy | 1. I feel confident that I can engage patients by asking how they feel since they have been admitted to hospital. 2. I feel that discussing with patients about their expectations of care after discharge comes easily to me. 3. I have confidence in my ability to convey courtesy and respect to all patients. |
| D5 Beliefs about consequences | Outcome expectancies | 1. I always introduce myself to patients and their loved ones to show my interest in them as individuals beyond the illness 2. I ask patients about their anxieties/fears about their health condition to provide personalised care. 3. I discuss with patients about their care expectations at discharge to increase treatment adherence. |
| D6 Motivation and goals | Action planning | 1. I thoroughly review the patient’s medical record to be well-prepared before each planned interaction with them 2. I plan specific questions to ask patients about their anxieties/fear to understand how they feel about their health condition. 3. I plan the opportune time to speak with patients about their expectations of post-discharge care. |
|  | Priority | 1. There are more urgent assigned duties that I need to complete than having detailed discussion with patients about their discharge plan. 2. I find that completing other assigned duties often take a higher priority than reviewing patients’ medical information before speaking with them. 3. When planning for discharge, there are other duties more pressing than speaking to patients about their anxieties/fears about their care. |
| D5 Beliefs about consequences | Reinforcement | 1. When patients feel that I am working towards their care goals, I get recognition from patients for providing a good care experience. 2. When I treat patients with courtesy and respect, I am repaid with the same treatment. 3. When I speak with patients about their anxieties/fears with their health condition, they tell me that is appreciated. |
